# Potential Benefits of Multimedia-Based Home Catheter Management Education in Patients With Peripherally Inserted Central Catheters: Systematic Review

**DOI:** 10.2196/17899

**Published:** 2020-12-10

**Authors:** Kija Malale, Jili Fu, William Nelson, Helena Marco Gemuhay, Xiuni Gan, Zhechuan Mei

**Affiliations:** 1 Department of Nursing The Second Affiliated Hospital of Chongqing Medical University Chongqing China; 2 Daping Hospital Army Medical University Chongqing China; 3 Department of Endocrinology and Metabolism The Second Affiliated Hospital of Chongqing Medical University Chongqing China; 4 School of Public Health and Social Sciences Muhimbili University of Health and Allied Sciences Dar es Salaam United Republic of Tanzania; 5 School of Nursing St John's University of Tanzania Dodoma United Republic of Tanzania; 6 Department of Gastroenterology The Second Hospital of Chongqing Medical University Chongqing China

**Keywords:** home catheter management, multimedia-based education, peripherally inserted central catheter

## Abstract

**Background:**

In recent years, there have been many suggestions to use multimedia as a strategy to fully meet the educational needs of patients with peripherally inserted central catheters. However, the potential benefits remain unreliable in the literature.

**Objective:**

In this study, we identified the potential benefits of multimedia-based home catheter management education in patients with peripherally inserted central catheters and discussed the clinical implications.

**Methods:**

We performed systematic searches of the PubMed, Cochrane Library, Embase Ovid, Medline, BioMed Central-cancer (BMC-cancer), ScienceDirect, and Google Scholar databases without date constraints until November 30, 2019. The methodological quality of the eligible studies was appraised using the Cochrane risk of bias tool. Narrative synthesis of the study findings was conducted.

**Results:**

A total of 6 intervention studies met the inclusion criteria, including 3 randomized controlled trials and 3 case-control studies/quasi-experimental studies. The studies included a total of 355 subjects, including a total of 175 in the multimedia groups and 180 in the control groups. We identified 4 potential benefits to patients: (1) improved knowledge, (2) increased satisfaction, (3) reduced incidence of catheter-related complications, and (4) reduced number of cases of delayed care after complications.

**Conclusions:**

The current systematic review highlights the potential benefits of multimedia-based home catheter management education for patients with peripherally inserted central catheters.

## Introduction

### Background

With the innovation of vascular access, the application of peripherally inserted central catheters is transforming care for patients treated with long-term infusion therapy in home care [1-3]. A peripherally inserted central catheter is a vascular access device that is inserted into the superficial or deep veins of the upper or lower extremities and advances the distal third of the superior vena cava or the proximal third of the inferior vena cava [1,2]. These catheters are often used in cancer patients, especially those requiring long-term infusion therapy [2]. Compared to the use of central venous catheters, infusion of vesicant/irritants and hypertonic solutions using peripherally inserted central catheters is safer, less costly, and more reliable [1]. During treatment, patients can live at home with a catheter for weeks to months [4,5].

Previous studies have shown that home catheter management education before catheterization plays an important role not only in preventing catheter-related complications, but also in improving catheter retention [6-9]. In order to maximize the clinical benefits of peripherally inserted central catheter services, home catheter management education is provided to each patient prior to catheterization. The information provided includes how to flush the catheter, when to change the dressing, when to clean the catheter, how to identify signs and symptoms of catheter-related complications, how to identify high-risk behaviors, and how to check if the catheter is inserted correctly [10,11]. However, the main challenge remaining is the lack of educational approaches that could effectively meet the educational needs of patients. The traditional didactic approach, commonly used in clinical settings around the world, is considered ineffective [1,3,12-16]. Patients complain that they get too little or too much information, which can be unhelpful, scary, technical, and hard to understand [12-14,16,17]. Patients whose educational needs are not met can become distressed, dissatisfied with the care provided, and have a diminished quality of life [18-22]. Sometimes they demand the catheter to be removed immediately after insertion [15]. In addition, other studies have found an increased incidence of catheter-related complications, including infection, catheter obstruction, thrombosis, and catheter displacement [23]. These hurdles stress the need for a new approach to effectively meet the educational needs of patients.

Technological innovation is not lagging in responding to the education challenges of patients, especially patients with peripherally inserted central catheters [3,6,9,10,23]. Patients benefit from the adaptability of technology and can use it to start, stop, and resume learning at their own pace. In recent years, some centers have experimented with technological innovations in the form of multimedia (eg, text, audio, images, animation, video, and voiceover interactive PowerPoint) to educate patients [9-11,24]. However, the potential benefits of multimedia-based education remain unclear. To our understanding, the existing literature is conflicting, which can confuse clinicians when choosing effective approaches to meet patients' educational needs [9-11,25]. Moreover, during our literature search, we found no systematic review or meta-analysis study that could synthesize the existing evidence.

### Objective

The main objective of this review was to determine the potential benefits of multimedia-based home catheter management education in patients with peripherally inserted central catheters and to discuss the clinical implications.

## Methods

### Design

The review was conducted in accordance with the Preferred Reporting Items for Systematic Reviews and Meta-analyses (PRISMA) guidelines [26]. A detailed protocol for the study was formulated prior to data collection.

### Search Strategy and Selection Criteria

The search strategy was conducted using MeSH key terms and their respective original words. We searched for the following: “Patient Education as Topic“ [Majr] OR “Patient Education” OR “Health Education” [Majr] OR “Health Education” AND “Catheterization, Peripheral” [Majr] OR “Peripherally Inserted Central Catheter” OR “PICC”. Electronic databases such as PubMed, Cochrane Library, Embase Ovid, Medline, BioMed Central-cancer (BMC-cancer), ScienceDirect, and Google Scholar were searched without date constraints until November 30, 2019. The review was limited to published articles written in English. Additional sources were identified by hand-searching reference lists of relevant study using Google search. Two independent researchers conducted the search in accordance with the set criteria.

Studies were included based on the following set of inclusion criteria: (1) an interventional study with two or more comparative groups; (2) involved subjects who had been prescribed or had already been installed with a peripherally inserted central catheter; (3) the educational approaches (interventions) employed were either didactic sessions (orally presented) as a standard (control) or were supplemented with multimedia (text, audio, image, animation, video, or voiceover interactive PowerPoint) for the test group; (4) the study reported one or more outcomes. Studies were excluded if (1) they included subjects aged <18 years (children); (2) they had unclear educational interventions; (3) subjects received interventions of a similar kind prior the study or different interventions during the follow-up period; or (4) they included critically ill patients (See Multimedia Appendix 1: PubMed search results summary).

### Quality Appraisal

We used the Cochrane risk assessment tool [27] to assess the risk of bias in randomized controlled trials and the ROBIN-1 tool [28] to assess the risk of bias in nonrandomized studies. In randomized controlled trials, the risk of bias was judged to be low risk, high risk, or unclear risk in each of these 6 aspects: (1) random sequence generation (selection bias), (2) allocation concealment (selection bias), (3) blinding of participants and personnel (performance bias), (4) blinding of outcome assessment (detection bias), (5) risk of attrition and reporting bias, and (6) any other sources of bias. In the nonrandomized studies, the risk of bias in each study was judged to be low risk, moderate risk, serious risk, critical risk, or no information in each of these seven aspects: (1) bias due to confounding, (2) bias in selection of participants into the study, (3) bias in classification of interventions, (4) bias due to deviations from intended interventions, (5) bias due to missing data, (6) bias in measurement of outcomes, and (7) bias in selection of the reported results. Two independent evaluators assessed the methodological quality of each study. If their results were in conflict, a third evaluator was included. The strength of evidence per outcome was rated using the Grading of Recommendations Assessment, Development, and Evaluation (GRADE) software [29].

### Data Abstraction and Synthesis

Data were extracted by 2 independent data collectors following a discussion of any conflicting results. Information pertaining to author name(s), year, country, study aim/outcomes, design, intervention type, sample size, and key findings in each study were extracted. Methodological quality of the included studies was uneven, which limited the meta-analysis. We chose to report the findings across the studies using a narrative synthesis format.

## Results

### Search Results

**Figure 1:**
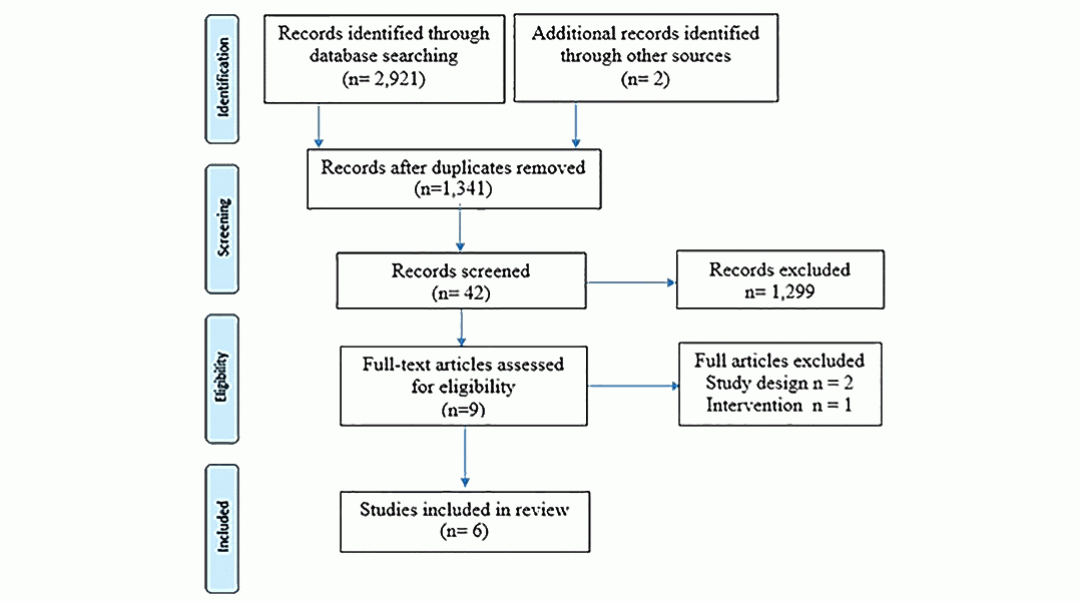
PRISMA flow chart for screening and selecting studies.

A search produced 2923 studies: PubMed (222), Cochrane Library (10), Embase Ovid (23), Medline (10), BMC-Cancer (1), ScienceDirect (281), and Google Scholar (2374). The other 2 studies come from the Google search engine. After removing duplicates and screening, 9 studies were considered potentially eligible [6-11,24,25,30]. After further evaluation, 6 studies met our inclusion criteria [8-11,24,25]. Two studies were excluded because of unclear educational interventions [6,7], and the other because of unrelated design [30].Three studies came from the United States [9,10,25], 1 from Canada [24], 1 from France [8], and 1 from Italy [11]. In addition, 3 studies were randomized controlled trials [9,11,24], and 3 were quasi-experimental/case-control studies [8,10,25]. Figure 1 shows the PRISMA flow chart for screening and selecting studies.

### Study Characteristics

The main characteristics of the 6 eligible studies are shown in Table 1. These studies included a total of 355 subjects, including 175 (49%) in the multimedia-based education group. The number of subjects in each study ranged from 11 [10] to 130 [25]. Follow-up for each study ranged from 1 day to 10 months. Different studies use different multimedia formats. Of the 6 studies, 4 used multimedia in the form of video [10,11,24,25], 1 used voiceover interactive PowerPoint [9], and 1 used pictures [8]. Eligible studies reported various outcomes, including patient understanding [24], patient comprehension [8], patient knowledge [9,11,25], patient satisfaction [8,10,24,25], catheter-related complications [8-10], and seeking medical care after complications occurred [8,9]. For the purposes of the current review, the terms “patient understanding,” “patient knowledge,” and “patient understanding” are merged into “patient knowledge.” Of the 5 studies that reported on patient knowledge, 4 used questionnaires [9,11,24,25], and 1 used the repeat-back method [8]. In the studies, patient satisfaction was evaluated by either questionnaire [10,24], the Likert-scale [8,25], or observation [11]. Catheter-related complications and seeking medical care after complications occurred were assessed by counting the number of cases of complications and the number of calls to health care providers/the number cases of delayed medical care after complications occurred.

**Table 1:** Eligible study characteristics.

Authors, country	Outcome(s)	Design	Sample size, n (control sample size)	Interventions
Veyrier et al [8], France	Comprehension, satisfaction, occurrence of adverse events, and seeking medical care after adverse events	Prospective case-control	30 (17)	OP^a^ plus cartoon and card game versus OP alone (control)
Fusco et al [11], Italy	Knowledge	Randomized controlled trial	40 (27)	OP plus video presentation versus OP alone (control)
Emery et al [9], USA	Knowledge, catheter-related complications, and seeking medical care	Randomized controlled trial	51 (27)	OP plus VOIPP^b^ versus OP alone (control)
Bowers et al [24], Canada	Understanding and satisfaction	Randomized controlled trial	93 (44)	OP plus video presentation versus OP alone (control)
Petroulias [10], USA	Satisfaction and catheter-related complications	Quasi-experimental study	11 (national data)	OP plus video presentation versus OP alone (control)
Sowan et al [25], USA	Knowledge recall, retention, satisfaction with the consent process, and multimedia patient decision aids	Quasi-experimental study	130 (65)	OP plus multimedia patient decision aids versus OP alone (control)

^a^OP: oral presentation.

^b^VOIPP: voiceover interactive PowerPoint.

### Methodological Quality of Eligible Studies

#### Randomized Controlled Trials

Of the 3 randomized controlled trials, the risk of random sequence generation (selection bias) was considered low in 2 studies [11,24] and unclear in 1 [9]. Only 1 study [24] involved allocation concealment (selection bias). The risk of bias due to the blinding of participants and personnel (performance bias) was unknown for all of the studies. Blinding of outcome assessment (detection bias) was considered high-risk in 2 studies [9,11] and unclear in 1 study [24]. All studies had a low risk of attrition and reporting bias. The other sources of bias were also considered low-risk in all studies. Table 2 shows risk of bias summary for randomized controlled trials.

**Table 2:** Risk of bias summary for randomized controlled trials.

Author	Random sequence generation (selection bias)	Allocation concealment (selection bias)	Blinding of participants and personnel (performance bias)	Blinding of outcome assessment (detection bias)	Incomplete outcome data (attrition bias)	Selective reporting (reporting bias)	Other bias
Bowers et al [24]	✓	✓			✓	✓	
Emery et al [9]				×	✓	✓	
Fusco et al [11]	✓			×	✓	✓	

#### Nonrandomized Controlled Trials

Of the nonrandomized studies, 2 of the 3 [8,10] were identified as having serious confounding-bias, and 1 as having moderate confounding bias [25]. Two of the studies [8,25] had a moderate selection bias risk, and 1 [10] had a critical risk of bias. In 2 studies [8,10], the risk of bias in the intervention classification was considered moderate, while in the other study [25] it was considered low. The risk of bias due to missing data was considered low in 2 studies [10,25] and critical in the third study [8]. The risk of bias in outcome measures was considered low in 2 studies [10,25] and critical in the other. In addition, bias in selection of the reported results was considered low risk in all 3 studies. The overall risk of bias was considered critical in 2 studies [8,10] and moderate in the other study [25]. Table 3 shows risk of bias summary for nonrandomized controlled trials.

**Table 3:** Risk of bias summary for nonrandomized controlled trials.

Authors	Bias due to confounding	Bias in selection of participant	Bias in classification of intervention	Bias due to missing data	Bias in measurement of outcome	Bias in selection of the reported results	Overall results
Petroulias [10]	Serious	Critical	Moderate	low	Low	Low	Critical
Veyrier et al [8]	Serious	Moderate	Moderate	Critical	Moderate	Low	Critical
Sowan et al [25]	Moderate	Moderate	Low	Low	Low	Low	moderate

### Synthesis

#### Overview

After a comprehensive narrative synthesis of the eligible key findings, 4 key aspects were identified as potential benefits of multimedia-based home catheter management education for patients with peripherally inserted central catheters. Key aspects identified include (1) knowledge improvement; (2) satisfaction with the information and services provided; (3) incidence of catheter-related complications; and (4) improved seeking of medical care after complications occur. The strength of evidence for each principal outcome was also measured (see Multimedia Appendix 2: Principal findings summary and evidence strength of each outcome).

#### Patient Knowledge Improvement

Five studies evaluated the knowledge of patients after intervention [8,9,11,24,25]. However, due to the different assessment tools and educational contents across studies, meta-analysis could not be conducted. Emery and colleagues [9] used a self-administered questionnaire containing 6 items. Items included (1) proper length of time to clean the end connector, (2) proper length of time to wash hands before initiating parenteral nutrition procedure, (3) preferred solution to clean work surfaces, (4) the first step to take when a crack or hole is found in a catheter, (5) steps that do not require notification of the Home Parenteral Nutrition service, and (6) important steps in preventing accidental removal of the catheter. Bowers and colleagues [24] used a self-administered questionnaire containing 5 true or false questions based on a particular procedure as well as the nature, risks, benefits, and procedures of catheter installation. Fusco [11] and colleagues used a self-administered questionnaire consisted of a series of questions, including closed, dichotomous, multiple choice, and open questions[11]. Veyrier and colleagues [8] used a repeat-back technique to assess how well patients understood what they had learned in previous discussions. Sowan and colleagues [25] used a structured questionnaire with 19 multiple choice and true or false questions covering the procedure indications, benefits, contraindications, insertion site, complications, and patient and health care team roles in the care and safety of a peripherally inserted central catheter. Despite differences in research methods, patient knowledge improved in all studies regardless of the educational intervention. Nevertheless, the improvement of knowledge in the multimedia groups was better than that in the control groups [8,11,24,25]. Table 4 summarizes the effects of multimedia-based education on patient knowledge improvement.

**Table 4:** Effect of multimedia-based education on patient knowledge improvement.

Author, country	Design	Interventions	Follow-up (days)	Assessment tool	Narrative summary of results
Veyrier et al [8], France	Prospective case-control	OP^a^ plus cartoon and card game versus OP alone (control)	1-2	Repeat-back	The overall adverse events comprehension score of the test group was higher than that of the control group.
Fusco et al [11], Italy	Randomized controlled trial	OP plus video presentation versus OP alone (control)	1	Questionnaire	Compared with the OP group, the overall peripherally inserted central catheter management knowledge score of the test group improved more.
Emery et al [9], USA	Randomized controlled trial	OP plus VOIPP^b^ versus OP alone (control)	1-10	Questionnaire	Catheter care knowledge scores increased in similar ways in each group immediately after the intervention and 7-10 days after the intervention.
Bowers et al [24], Canada	Randomized controlled trial	OP plus video presentation versus OP alone (control)	1	Questionnaire	The test group was significantly better than the control group in peripherally inserted central catheter insertion procedure comprehension score.
Sowan et al [25], USA	Quasi-experimental study	OP plus multimedia patient decision aids versus OP alone (control)	1-2	Questionnaire	Compared with the control group, the multimedia group scored 2 points higher in knowledge recall and retention with the consent process.

^a^OP: oral presentation.

^b^VOIPP: voiceover interactive PowerPoint.

#### Patient Satisfaction With the Information and Services Provided

Four studies assessed patient satisfaction with the information and services provided [8,10,24,25], but meta-analysis was not possible due to the use of different tools for evaluation. Veyrier and colleagues [8] used a 4-point Likert-type scale that ranged from 4 (very satisfied) to 1 (dissatisfied). Sowan and colleagues [25] used a 5-point Likert-type scale that ranged from 5 (very satisfied) to 1 (very unsatisfied). Both Petroulias [10] and Bowers and colleagues [24] used anecdotes and questionnaires to assess patient satisfaction with the information provided, respectively. The results of all studies indicated that patients were satisfied or very satisfied with the information and multimedia devices. However, some patients in the multimedia group showed increased anxiety, especially those with a history of complications [8]. Table 5 summarizes effect of multimedia-based education on patient satisfaction.

**Table 5:** Effect of multimedia-based education on patient satisfaction improvement.

Author, country	Design	Interventions	Follow-up (days)	Assessment tool	Results summary
Veyrier et al [8], France	Prospective case-control	OP^a^ plus cartoon and card game versus OP alone (control)	1-2	Likert-scale	Patients in the test group were satisfied or very satisfied with the intervention and the quality of the documents provided.
Bowers et al [24], Canada	Quasi-experimental study	OP plus video presentation versus OP alone (control)	1	Questionnaire	The overall satisfaction of patients in the test group was significantly higher than that in the control group.
Petroulias [10], USA	Quasi-experimental study	OP plus video presentation versus OP alone (national data)	42	Questionnaire	Overall satisfaction with learning how to maintain the peripherally inserted central catheter at home improved after the video presentation.
Sowan et al [25], USA	Quasi-experimental study	OP plus multimedia patient decision aids versus OP alone (control)	1-2	Questionnaire	Overall patient satisfaction scores improved in a similar way between groups.

^a^OP: oral presentation.

#### Incidence of Catheter-Related Complications

Three studies assessed the incidence of catheter-related complications [8-10], but meta-analysis was not possible due to the different study designs used. There was a randomized controlled trial [9], a case-control study [8] and a quasi-experimental study [10]. In addition, the follow-up period varied across studies (42 days [10], 90 days [9], 10 months [8]). However, in all the studies, the incidence of catheter-related complications was slightly lower in the multimedia group than in the control group. The common complications observed in the multimedia group were infection, thrombosis, and displacement. Table 6 summarizes the effect of multimedia-based education on incidence of catheter-related complications.

**Table 6:** Effect of multimedia-based education on incidence of catheter-related complications.

Author, country	Design	Interventions	Follow-up (days)	Assessment tool	Results summary
Veyrier et al [8], France	Prospective case-control	OP^a^ plus cartoon and card game versus OP alone (control)	305	Medical records	The risk of adverse events in the multimedia group was 38.5% lower than that in the control group.
Emery et al [9], USA	Randomized controlled trial	OP plus VOIPP^b^ versus OP alone (control)	90	Questionnaire	All-cause readmissions in the VOIPP group was 14.4% lower than that in control group. The incidence of catheter-related blood stream infections in the VOIPP group was 10.6% lower than that in control group.
Petroulias [10], USA	Quasi-experimental study	OP plus video presentation versus OP alone (national data)	42	Medical records	The incidence of catheter occlusion was 0% in the video group and 14%-36% nationally (national data).

^a^OP: oral presentation.

^b^VOIPP: voiceover interactive PowerPoint.

#### Seeking Medical Care After the Occurrence of Complications

Two studies evaluated the effect of intervention on seeking medical care after the occurrence of complications [8,9], but meta-analysis was not possible due to different reporting methods. Emery and colleagues [9] used the number of calls received by health care providers, while Veyrier and colleagues [8] used the number of delayed visits after complications occurred. Communication between patients and medical staff in the multimedia group was improved, and there were fewer cases of delayed treatment after complications. The number of calls to health care providers in the multimedia group increased significantly compared to the control group. Patients in the multimedia group were more likely than those in the control group to report or seek clarification from their healthcare provider if problems arose [9]. Likewise, the number of cases of delayed medical treatment after complications decreased significantly in the multimedia group compared with the control group [8]. Table 7 summarizes the effect of multimedia-based education on seeking medical care after the occurrence of complications.

**Table 7:** Effect of multimedia-based education on seeking medical care after the occurrence of complications.

Author, country	Design	Interventions	Follow-up (days)	Assessment tool	Results summary
Veyrier et al [8], France	Prospective case-control	OP^a^ plus cartoon and card game versus OP alone (control)	305	Medical records	Risk of delay to sick medical care after adverse event occur was lower in the test group compared to the control group (0% versus 100%).
Emery et al [9], USA	Randomized controlled trial	OP plus VOIPP^b^ versus OP alone (control)	90	Medical records	Patients in the test group were more likely than those in the control group to report or seek clarification from their healthcare provider if problems arose.

^a^OP: oral presentation.

^b^VOIPP: voiceover interactive PowerPoint.

## Discussion

### Principal Findings

In recent years, there have been many suggestions to provide multimedia-based home catheter management education for patients with peripherally inserted central catheters [9,11,24].Our study determined the potential benefits of multimedia-based home catheter management education for patients with peripherally inserted central catheters and discusses the clinical implications. We identified 4 major potential benefits for patients: (1) improved knowledge, (2) increased satisfaction, (3) reduced incidence of catheter-related complications, and (4) reduced number of cases of delayed medical care after complications. The generalizability of the findings is limited due to the low methodological quality, small number of studies, and the use of multiple forms of multimedia across studies.

Multimedia-based education approach can improve home catheter management knowledge of patients living with peripherally inserted central catheter. In this review, 4 out of 5 studies reported that multimedia-based education had a beneficial impact on patients by improving their knowledge of peripherally inserted central catheter management [8,11,24,25]. Bowers and colleagues [24] found that patients in the multimedia presentation group scored significantly better on comprehension than those in the control group. Fusco and colleagues [11] found that the overall knowledge score of the video supplement group was significantly higher than that of the interview group or the interview plus brochure group. Veyrier and colleagues [8] found that patients' comprehension improved significantly when they were given two pharmaceutical consultations using cartoons and a card game demonstration, compared with a control group. Furthermore, Sowan and colleagues [25] found that patients in the multimedia patient decision aids group had higher levels of knowledge recall and retention than patients in the control group who were given information orally. This is likely because traditional peripherally inserted central catheter management education relies on patients passively acquiring knowledge through didactic sessions and instructional manuals, whereas most patients actively request to learn at their own pace and view pictures related to the information presented. Patients, especially those diagnosed with cancer, are often intellectually challenged by the diagnosis of the disease and pay little attention to the verbal information provided by medical staff. Therefore, adopting flexible learning approaches, such as multimedia-based education, can help patients to resume learning more frequently when they are in a good mood. However, Emery and colleagues [9] found no statistically significant difference in overall knowledge scores between the voiceover interactive PowerPoint supplement group and the control group immediately after the intervention or 7 to 10 days later. The variability of the results of later studies may be due to the mix of educational content without considering the impact on the outcome of the intervention. In the study by Emery and colleagues, participants were given information about the tunneled catheter and peripherally inserted central catheter, but the authors did not explain how this confounding factor was considered in the questionnaire and data analysis [9].

Multimedia-based education can improve patients' satisfaction with information and services provided. In the current review, 4 studies evaluated patient satisfaction with information and services provided [8,10,24,25], but meta-analysis was excluded due to the use of different assessment tools. Nevertheless, all studies showed that patients were satisfied with the peripherally inserted central catheter information and services. Bowers and colleagues [24] found that satisfaction with peripherally inserted central catheter information and services during the informed consent process was significantly higher in the multimedia presentation group than in the control group. Similarly, patients in the multimedia groups were more satisfied with peripherally inserted central catheter information and devices [8,10,25]. These improvements may be related to multimedia technology, which allows patients to access more dynamic and easily understood information at their fingertips, rather than the traditional didactically taught approach, which requires patients to see a health care provider. However, continued access to some information about peripherally inserted central catheter, such as images depicting the location of the catheter in the heart, has been reported to increase anxiety, especially in patients with a history of complications [8]. The results further emphasize the need to customize peripherally inserted central catheter management information based on patient preferences and needs, which is of great value in improving patient satisfaction with the information and services provided.

Multimedia-based education can moderately reduce the incidence of catheter-related complications. Three studies evaluated the effect of multimedia-based education on catheter-related complications [8-10], but meta-analysis was not possible due to different study designs. All studies reported a slight decrease in catheter-related complications incidence in the multimedia-based peripherally inserted central catheter management education group compared to the control group. This may be because in order for patients to be able to make decisions for the peripherally inserted central catheter and to accurately implement the recommendations of their healthcare provider, they need to be fully aware of catheter management skills. Compared with the traditional didactic education approach, the peripherally inserted central catheter management education based on multimedia not only improves the knowledge level of patients, but also improves their satisfaction with information and services provided. Petroulias [10] successfully reduced the occlusion rate of patients by using electronic tablets loaded with video to educate patients on how to flush peripherally inserted central catheter line. Patients received FaceTime training and watched videos showing the 10 steps of flushing. The results showed that the occlusion rate of peripherally inserted central catheters was significantly lower than when traditional teaching methods were used. Likewise, Emery and colleagues [9] found that the voiceover interactive PowerPoint group had a 10.6% lower incidence of catheter-related blood stream infections than the control group.

Multimedia-based education can reduce medical care delays when complications occur. Two studies assessed the risk of delay in seeking medical care when complications occurred [8,9], but due to different reporting methods, meta-analysis was not possible. Emery and colleagues [9] found that patients in the voiceover interactive PowerPoint group communicated with their health care provider more frequently than patients in the control group. Most calls were for information about the catheter itself, including its site problem, occlusion or rupture, and dressing problem. Similarly, Veyrier and colleagues [8] found that the strip cartoon and card game group had a lower risk of delayed medical care after an adverse event than the control group. This is likely because better communication between patients and healthcare providers not only makes it easier for patients to follow-up, but also clarifies problems they still do not understand. In addition, the improvement of patients’ knowledge of peripherally inserted central catheter management is also considered to enable patients to pay more attention to or observe the changes in the catheter or themselves and report to the medical staff in a timely manner.

### Strengths and Limitations

Our study has its strengths and limitations. The main strengths of our study include the use of a systematic approach to search for studies, the use of well-known electronic databases, comprehensive analysis of eligible study results, and extensive discussion of current study findings. However, the generalizability of current study findings is limited by several factors, including the small number of eligible studies, small sample size in some of the eligible studies, the low methodological quality of studies, the unreliable tools used to measure outcomes, the lack of long-term follow-up studies in particular to knowledge, and studies using different types of multimedia.

### Conclusions

In this study, the potential benefits of multimedia-based home catheter management education for patients with peripherally inserted central catheter were highlighted, and the clinical implications were discussed. The findings revealed that peripherally inserted central catheter management education through multimedia-based presentation not only improved the knowledge level and satisfaction of patients, but also reduced the incidence of catheter-related complications and reduced the medical care delay when complications occurred. Due to the low methodological quality and number of included studies and the use of multiple multimedia formats, the generalizability of the findings is limited. Further review, including original studies of high methodological quality, is needed to confirm the current findings.

## Appendix

Multimedia Appendix 1 PubMed search results summary.

Multimedia Appendix 2 Principal findings summary and evidence strength of each outcome.
