# Supplementary material for: Potential Benefits of Multimedia-Based Home Catheter Management Education in Patients With Peripherally Inserted Central Catheters: Systematic Review
Source: J Med Internet Res. 2020 Dec 10;22(12):e17899. doi: 10.2196/17899 (PMC7759441; doi:10.2196/17899)
Supplement: Multimedia Appendix 1 [file jmir_v22i12e17899_app1.pdf]

Multimedia Appendix 1: PubMed search results summary

S/N	Key word/MeSH term	Search Results
**A**	“Patient Education”[Majr]	39,273
**B**	“Patient Education”	100,645
**C**	“Health Education"[Majr]	142,662
**D**	“Health Education”	704,804
**E**	“**A**” OR “**B**” OR “**C**”	713,212
**F**	"Catheterization, Peripheral"[Majr]	7,985
**G**	“Peripherally Inserted Central Catheter”	2,301
**H**	“PICC”	1,209
**I**	“**F**” OR “**G**” OR “**H**”	9,613
**J**	“**E**” AND “**I**”	222
