# Supplementary material for: Potential Benefits of Multimedia-Based Home Catheter Management Education in Patients With Peripherally Inserted Central Catheters: Systematic Review
Source: J Med Internet Res. 2020 Dec 10;22(12):e17899. doi: 10.2196/17899 (PMC7759441; doi:10.2196/17899)
Supplement: Multimedia Appendix 2 [file jmir_v22i12e17899_app2.pdf]

Multimedia Appendix 2: Principal findings summary and evidence strength of each outcome

Principal finding	Studies	Design		Strength of evidence
		RCT (n)	Observation study (n)	
• Multimedia-based PICC management education can improve patients' knowledge	[9,11,17,24,25]	3	2	very low
• Multimedia-based PICC management education can improve patients' satisfaction with information and services	[10,17,24,25]	1	3	very low
• Multimedia-based PICC management education can moderately reduce the incidence of catheter-related complications	[9,10,17]	1	2	very low
• Multimedia-based PICC management education can reduce medical care delay when complications occur	[9,17]	1	1	very low
